# Genome-Wide Identification and Expression Analysis of the *DA1* Gene Family in Sweet Potato and Its Two Diploid Relatives

**DOI:** 10.3390/ijms25053000

**Published:** 2024-03-05

**Authors:** Zelong Zhou, Jianzhi Huang, Yuehui Wang, Shixiang He, Jing Yang, Ying Wang, Wenxing Li, Yi Liu, Ran Xu, Yunhai Li, Lian Wu

**Affiliations:** 1 School of Breeding and Multiplication (Sanya Institute of Breeding and Multiplication), Hainan University, Sanya 572025, China; jingzhuangyouan@163.com (Z.Z.); huangjianzhi55@163.com (J.H.); 21220951310095@hainanu.edu.cn (Y.W.); 15289794097@163.com (S.H.); 17836227016@163.com (J.Y.); sgwy6106@163.com (Y.W.); liwenxingsichuang@163.com (W.L.); 15549421602@163.com (Y.L.); xuran@hainanu.edu.cn (R.X.); 2 State Key Laboratory of Plant Cell and Chromosome Engineering, CAS Centre for Excellence in Molecular Plant Biology, Institute of Genetics and Developmental Biology, Chinese Academy of Sciences, Beijing 100101, China; yhli@genetics.ac.cn

**Keywords:** sweet potato, organ size, *DA1-like* gene, qRT-PCR

## Abstract

The *DA1-like* gene family plays a crucial role in regulating seed and organ size in plants. The *DA1* gene family has been identified in several species but has not yet been reported in sweet potatoes. In this study, nine, eleven, and seven *DA1*s were identified in cultivated sweet potato (*Ipomoea batatas*, 2n = 6x = 90) and its two diploid wild relatives, *I. trifida* (2n = 2x = 30) and *I. triloba* (2n = 2x = 30), respectively. The *DA1* genes were classified into three subgroups based on their phylogenetic relationships with *Arabidopsis thaliana* and *Oryza sativa* (rice). Their protein physiological properties, chromosomal localization, phylogenetic relationships, gene structure, promoter cis-elements, and expression patterns were systematically analyzed. The qRT-PCR results showed that the expression levels of four genes, *IbDA1-1*, *IbDA1-3*, *IbDA1-6*, and *IbDA1-7*, were higher in the sweet potato leaves than in the roots, fiber roots, and stems. In our study, we provide a comprehensive comparison and further the knowledge of *DA1-like* genes in sweet potatoes, and provide a theoretical basis for functional studies.

## 1. Introduction

The sizes of plant seeds and organs are important yield traits. An important topic in developmental biology is size regulation. Multiple genes involved in seed size regulation have been identified in plants, including *DA1* [1], *SGD1* [2], *GS3* [3], *OsLG3* [4], *LGY3* [5], *GW5* [6], *GW2* [7], *GW8* [8], *qGL3* [9], *GS10* [10], *GL4* [11], *TGW2* [12], and *GS9* [13]. *DA1* is an important gene that regulates seed size. This gene encodes a ubiquitin receptor. A protein sequence analysis has indicated that *DA1* contains two typical ubiquitin interaction motifs (UIMs) and a single zinc-binding (LIM) domain. Through ubiquitination, ubiquitin proteins regulate transcription factors’ and regulatory proteins’ stability and activity, which is a critical regulatory function in the growth and determination of seed size [14].

The *DA1-like* gene family has been identified in several crops: four genes in rapeseed (*Brassica napus*) [15], eleven genes in soybean (*Glycine max*) [16], six genes in maize (*Zea mays* L.) [17], three genes in oat (*Avena sativa* L.) [18], twenty-one genes in cotton (*Gossypium* spp.) [19], and four genes in rice (*Oryza sativa* L.) [20]. The *DA1* gene was initially discovered to regulate the size of leaves, seeds, flowers, and siliques in *Arabidopsis thaliana*. Additionally, abscisic acid (ABA) upregulates *AtDA1* expression, thereby positively regulating seed size [1]. The *DA2* gene encodes a ring-type E3 ubiquitin ligase that interacts with DA1, thereby influencing seed size. Decreasing the expression level of *BnDA1* in rapeseed significantly increased seed size, yield, and biomass [15]. In rice, *HDR3*, as a UIM-type active ubiquitin receptor, increases its ubiquitination level by interacting with GW6a. This slows down the degradation of the 26S proteasome. This effect promotes the grain filling rate, which increases the final grain size and weight [21]. The interaction of TaDA1 and TaGW2, homologous genes of *Arabidopsis thaliana DA1*, has been found in wheat. Both genes regulate grain size and have a genetic additive effect. The excellent haplotypes *TaDA1-2A* and *TaGW2-6B* are associated with a high 1000-grain weight, and they also exhibit additive effects [22]. The upregulation of the *ZmDA1* or *ZmDAR1* genes promotes grain development and enlargement, leading to increased yield in maize. In addition, *ZmDR1* regulates starch synthesis, promotes starch accumulation, and enhances its quality [17]. Therefore, improving crop yield and quality can be achieved by varying the *DA1-like* gene family’s expression levels.

*Ipomoea batatas* (L.) Lam. is a type of sweet potato that belongs to the Convolvulaceae group. It is known for its high yield, strong adaptability, and wide cultivation areas, making it a significant staple crop and industrial raw material [23,24]. The completion of the genome sequencing of sweet potato (*I. batatas* (L.) Lam., 2n = B_1_B_1_B_2_B_2_B_2_B_2_ = 6x = 90) [25] and its two diploid species, *I. trifida* NCNSP0306 (2n = 2x = 30) and *I. triloba* NCNSP0323 (2n = 2x = 30) [26], has made it possible to analyze the sweet potato gene family using bioinformatics [27,28,29,30,31,32]. However, the *DA1-like* gene family in sweet potatoes has not been reported in these whole-genome studies.

In this study, we identified *DA1-like* genes in sweet potato and two diploid species at the whole-genome level. We examined the gene architectures, promoter cis-acting elements, expression patterns, and evolutionary relationships of these *DA1-like* genes in several tissues. Moreover, the expression profiles of particular *DA1-like* genes were confirmed using quantitative real-time polymerase chain reaction (qRT-PCR) tests in four different tissue sites. These results supply crucial information for examining the roles of *DA1-like* genes in sweet potatoes.

## 2. Results

### 2.1. Identification of DA1-like Genes in Sweet Potato and Two Diploid Relatives

We used three strategies to comprehensively identify all of the members of the *DA1-like* gene family in sweet potato and its two diploid relatives. There are 27 genes in the *DA1-like* gene family that have been identified. Respectively, in the sweet potato and its two diploid relatives, there are 9, 7, and 11 *DA1-like* family members. By sequencing these *DA1-like* genes, the physicochemical properties were determined in *I. batatas*. The *IbDA1* CDS lengths ranged from 471 bp (*IbDA1-9*) to 1992 bp (*IbDA1-2*). There were 157 amino acids in IbDA1 (IbDA1-9) and 664 amino acids in IbDA1-2; the MW values differed from 17.91 kDa to 74.59 kDa; and the isoelectric point values ranged from 5.09 (IbDA1-6) to 7.49 (IbDA1-2). Among all of the *DA1-like* genes, only IbDA1-2 had a PI value greater than seven, indicating that it was a basic protein. There were Thr, Ser, and Tyr phosphorylation sites on all of the IbDA1s. The aliphatic index varied from 64.49 (IbDA1-6) to 85.64 (IbDA1-9). IbDA1-2 and IbDA1-9 had aliphatic indices exceeding 85, suggesting thermophilia. The GRAVY scores of all of the IbDA1 proteins were less than 0, which indicates that they are hydrophilic proteins. The subcellular localization predictions indicated that IbDA1-1, IbDA1-3, IbDA1-6, IbDA1-7, and IbDA1-8 would be found in the nucleus; IbDA1-4, IbDA1-5, and IbDA1-9 would be detected in the cytoplasm; and IbDA1-2 would be detected in the endoplasmic reticulum (Table 1).

**Figure 1:**
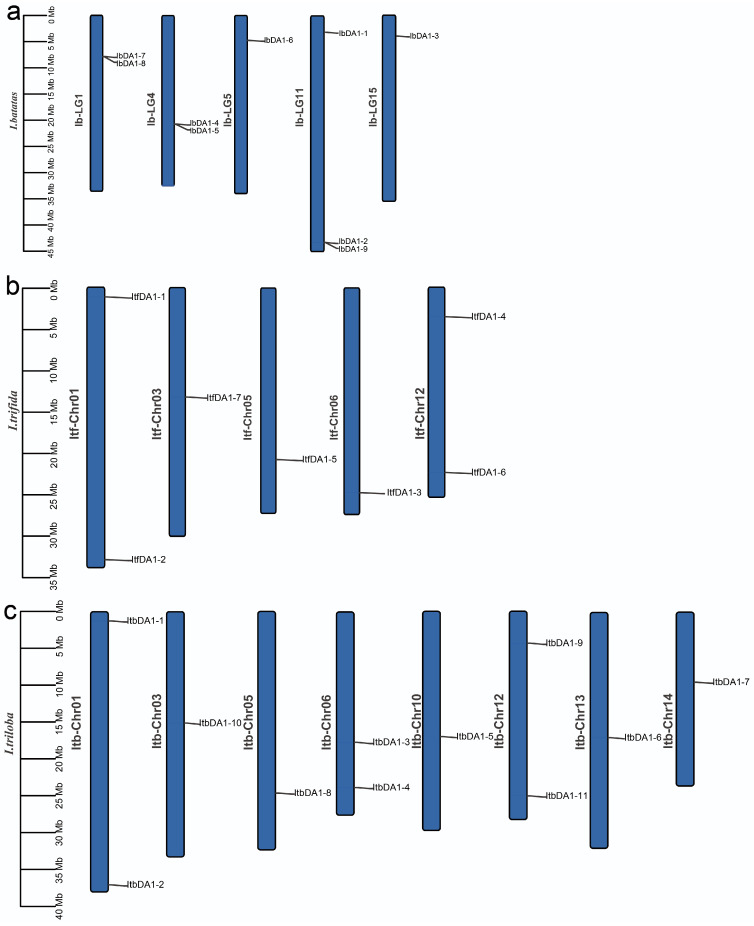
Chromosomal localization and distribution of *DA1-like* genes in *I. batatas* (**a**), *I. trifida* (**b**), and *I. triloba* (**c**).

A *DA1-like* gene can be found on chromosomes 5, 5, and 8 of *I. batatas*, *I. trifida*, and *I. triloba*, respectively. LG11 contained three *IbDA1*s (*IbDA1-1*, *-2*, and *9*), two were on LG1 (*IbDA1-7* and *-8*) and LG4 (*IbDA1-4* and *-5*), and LG5 (*IbDA1-6*) and LG15 (*IbDA1-3*) had one each in *I. batatas*. Two *ItfDA1*s were detected on LG1 (*ItfDA1-1* and *-2*) and LG12 (*ItfDA1-4* and *-6*), and one each on LG3 (*ItfDA1-7*), LG5 (*ItfDA1-5*), and LG6 (*ItfDA1-3*) in *I. trifida*. The *DA1-like* genes in *I. triloba* had the highest number of members, with one or two genes on each chromosome (Figure 1).

### 2.2. Evolutionary Analysis of the DA1-like Genes

**Figure 2:**
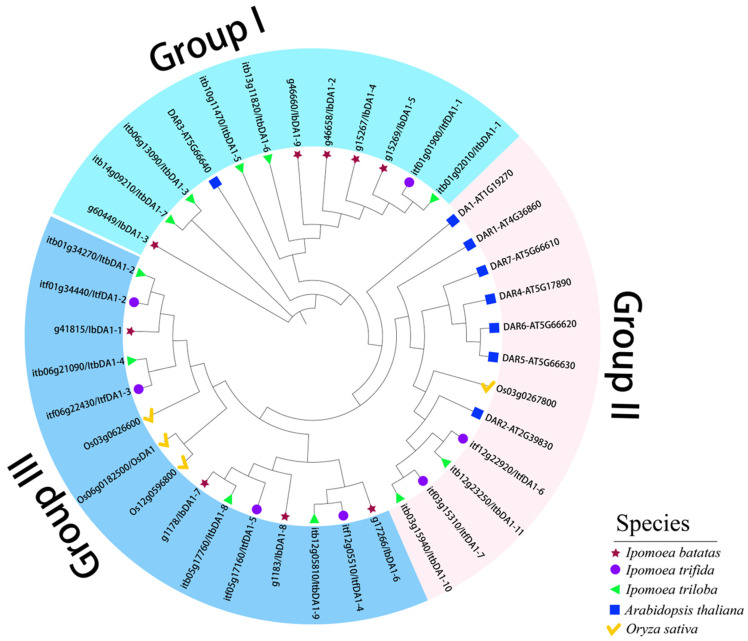
Phylogenetic analysis of the DA1 proteins from five plant species (i.e., *I. batatas*, *I. trifida*, *I. triloba*, *Arabidopsis thaliana*, and *Oryza sativa*). In total, 39 DA1s were divided into three subgroups (Groups I to III) according to their evolutionary distance. The red stars, purple circles, green triangles, blue rectangles, and yellow check mark represent the 9 IbDA1s in *I. batatas*, the 7 ItfDA1s in *I. trifida*, the 11 ItbDA1s in *I. triloba*, the 8 AtDA1s in *Arabidopsis thaliana*, and the 4 OsDA1s in *Oryza sativa*, respectively.

We constructed a phylogenetic tree for thirty-nine DA1 proteins (i.e., nine in *I. batatas*, seven in *I. trifida*, eleven in *I. triloba*, eight in *Arabidopsis thaliana*, and four in *Oryza sativa*) to study the evolutionary relationship of DA1 in *I. batatas*, *I. trifida*, *I. triloba*, *A. thaliana*, and *O. sativa* (Figure 2). Considering their evolutionary divergence, the *DA1* proteins found in sweet potatoes and their diploid relatives can be classified into three subclasses (Groups I to III). As a result, (for *I. batatas*, *I. trifida*, *I. triloba*, *A. thaliana*, and *O. sativa*) the DA1s were distributed in three groups: Group I (12: 5, 1, 5, 1, 0), Group II (12: 0, 2, 2, 7, 1), and Group III (15: 4, 4, 4, 0, 3).

### 2.3. An Analysis of the Conserved Motif and Exon and Intron Structures of DA1 Genes from Sweet Potato and Its Two Closest Relatives

**Figure 3:**
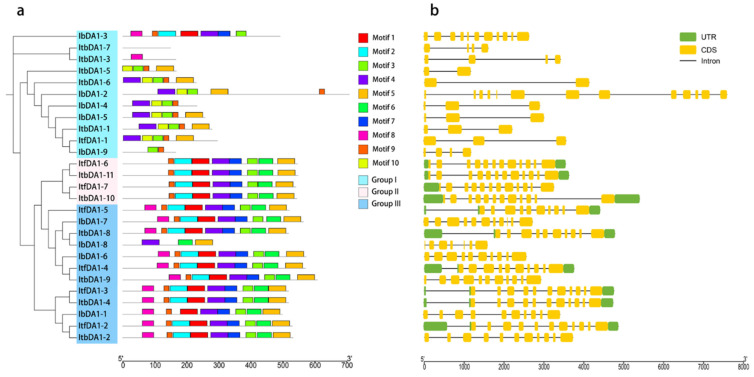
Conserved motifs and analysis of the exon–intron structure of the *DA1* family in *I. batatas*, *I. trifida*, and *I. triloba*. (**a**) The phylogenetic tree shows that the DA1s are distributed in three subgroups (left), and the ten conserved motifs are shown in different colors. (**b**) Exon–intron structures of DA1s. The green boxes, yellow boxes, and black lines represent the UTRs, exons, and introns, respectively.

An analysis of the sequence motifs in nine *IbDA1s*, seven *ItfDA1s*, and eleven *ItbDA1s* was performed using the MEME website. Ten highly conserved motifs were also identified. The number of motifs varied among the members, ranging from zero to nine. Over half of the members (16 *DA1*s) contained all ten motifs. Most of the *DA1*s contained eight of these conserved motifs, and only a few *DA1*s differed in their number and types of motifs, such as *IbDA1-9* (without a motif), *IbDA1-3* (lacking motifs 3, 6, and 10), and *IbDA1-1* (lacking motifs 4 and 10) (Figure 3a). We analyzed the exon and intron structures to comprehensively understand the structural characteristics of the DA1 genes. In the cultivated sweet potato, *IbDA1-2* had the largest number of exons (thirteen), whereas *IbDA1-4* and *IbDA1-5* had the smallest number of exons (three). Comparing the cultivated sweet potato and its two diploid wild relatives, we observed a substantial variation in the number of exons per gene, ranging from a maximum of thirteen to a minimum of only two exons. The *DA1-like* genes had distinct functional differences during evolution, according to these results (Figure 3b).

### 2.4. Sweet Potato and Its Two Diploid Relatives: A Cis-Element Analysis

**Figure 4:**
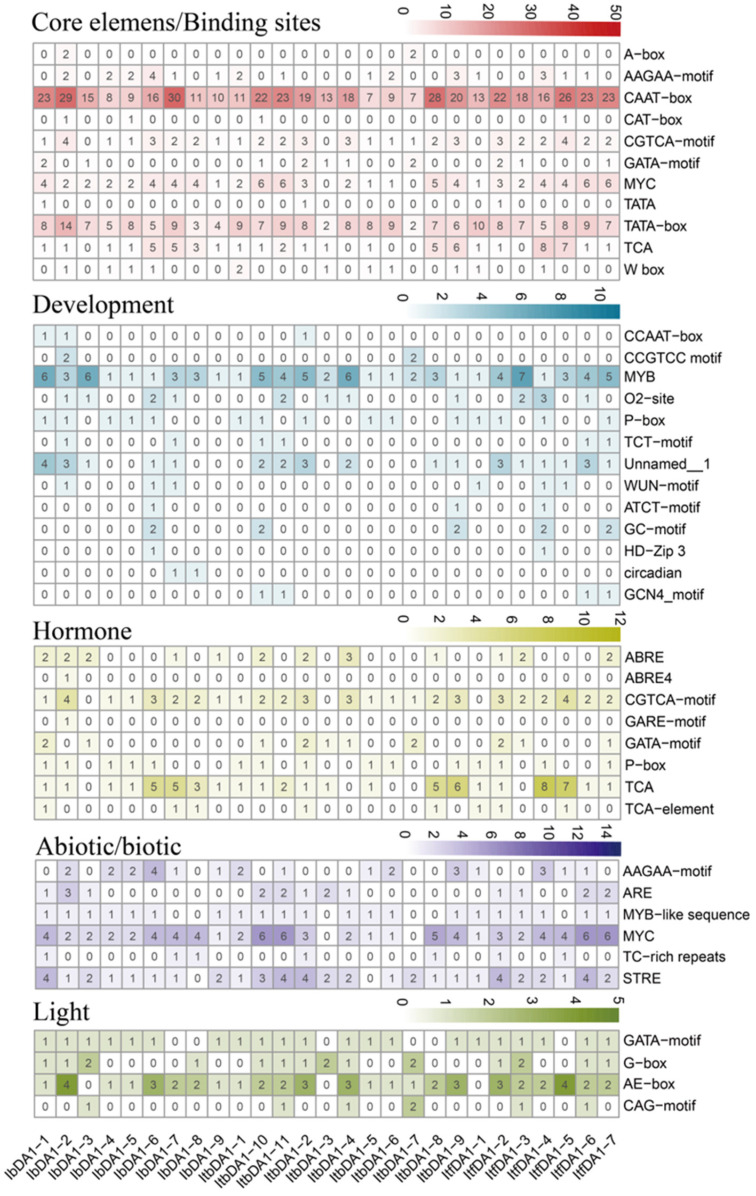
*Cis*-element analysis of DA1s in *I. batatas*, *I. trifida*, and *I. triloba*. The *cis*-elements were divided into five broad categories. The intensity of the different colors represents the number of *cis*-elements in the *DA1*s’ promoters.

*Cis*-elements participate in the regulation of gene expression. We analyzed the *cis*-regulatory elements present in the 2000 bp promoter regions of the *IbDA1*s, *ItbDA1*s, and *ItfDA1*s in order to investigate the molecular regulatory mechanisms of the *DA1*s. According to Figure 4, the elements can be grouped into five groups based on their predicted functions: core elements, binding sites, developmentally regulated elements, hormone-responsive elements, stress-responsive elements, and light-responsive elements.

All of the *IbDA1* members contained the core elements CAAT-box, TATA-box, MYC, and TCA; however, TCA was absent in *IbDA1-4*. The developmental transcription factor MYB was identified in all *IbDA1*s, whereas the majority of the *IbDA1*s contained P-box (*IbDA1-1*, *-2*, *-4*, *-5,* and *-6*) and O2-site (*IbDA1-2*, *-3*, *-6*, and *-7*) elements. Among these, *IbDA1-6* exhibited the highest diversity of developmental elements, including MYB, P-box, O2-site, WUN-motif, ATCT-motif, GC-motif, and HD-zip. In most of the *IbDA1*s, hormone- and light-responsive elements were observed, including CGTCA motifs for the MeJA themes, TCA themes for the SA themes, and AE-box motifs for the light motifs; however, these elements were absent from *IbDA1-3*. Additionally, the IbDA1 promoters exhibited a high abundance of drought-responsive MYC elements among the abiotic elements.

We analyzed two wild sweet potato relatives to clarify the characteristics of cis-acting elements in the *DA1*s’ promoter regions. Similar to the *IbDA1*s, the *ItbDA1*s and *ItfDA1*s have CAAT-boxes and TATA-boxes in their promoter regions as well as the developmental element MYB. In addition, most of the *ItbDA1*s and *ItfDA1*s had the developmental elements O2-site and P-box, the hormone elements CGTCA motif and TCA, the abiotic stress elements MYB, MYC, and STRE, and the light-responsive elements GATA-motif and AE-box.

### 2.5. Different Tissues Containing DA1-like Genes Analyzed via RNA-Seq

**Figure 5:**
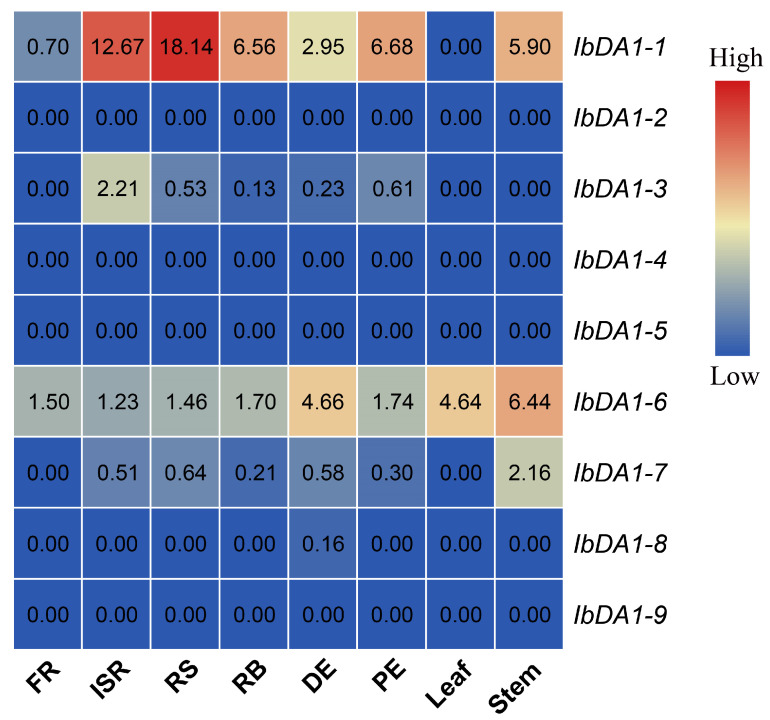
Expression pattern of the *DA1-like* genes in “Xushu 18” in different tissues. The eight tissues comprise stem, leaf, fibrous root (FR), initiated storage root (ISR), root stock (RS), root body (RB), distal end (DE), and proximal end (PE) tissues.

To identify the expression of the *DA1* gene in different tissues, we analyzed the published transcriptome data of “Xushu 18”. This was performed to clarify the potential function of the *DA1* gene in sweet potatoes. A heat map was created using fragments per kilobase of the exon model per million mapped fragments (FPKM). Usually, a gene’s FPKM value is greater than 1, indicating its expressibility and functionality. In this study, the FPKM of *IbDA1-6* in the sweet potatoes was greater than 1 in eight different tissues, indicating that *IbDA1-6* is expressible and functional. *IbDA1-1* was highly expressed in six tissues but not in the fibrous root (FR) or leaf. The FPKM values of *IbDA1-2*, *-3*, *-4*, *-7*, *-8*, and *-9* were almost all less than 1 in the eight sweet potato tissues, indicating that they were non-functional (Figure 5).

**Figure 6:**
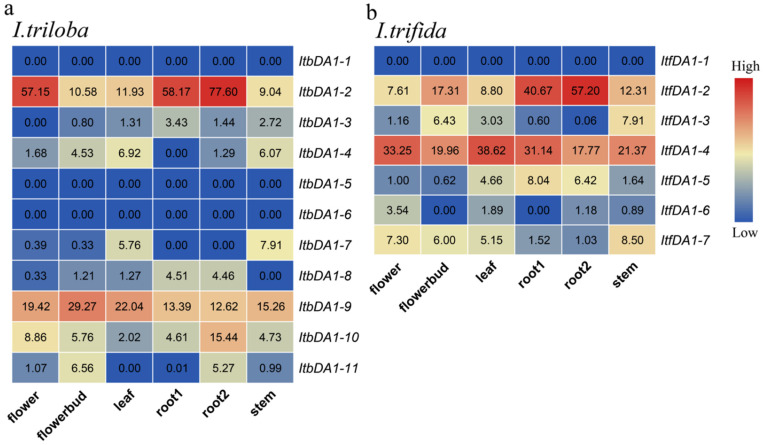
Gene expression patterns of *ItbDA1*s (**a**) and *ItfDA1*s (**b**) in different organs (root 1, root 2, stem, leaf, flower, and flower bud).

*DA1* expression was also investigated in *I. trifida* and *I. triloba* based on RNA-seq data collected from six tissues (flowers, flower buds, leaves, root 1, root 2, and stem). In *I. triloba*, a total of five *ItbDA1*s were highly expressed in the flowers: *ItbDA1-2*, *-4*, *-9*, *-10*, and *-11*. The flower buds expressed high levels of six ItbDA1s (i.e., ItbDA1-2, -4, -8, -9, -10, and -11). In the leaves, *ItbDA1-2*, *-3*, *-4*, *-7*, *-8*, *-9*, and *-10* were highly expressed. There were five ItbDA1s (*ItbDA1-2*, *-3*, *-8*, *-9*, and *-10*) found at high levels of expression in the roots (root 1 and root 2). In the leaves, six ItbDA1s were highly expressed *(ItbDA1-2*, *-3*, *-4*, *-7*, *-9*, and *10*). Moreover, three ItbDA1s (i.e., *ItbDA1-1*, *5*, and *6*) showed low expression levels in all of the tissues (Figure 6a).

In all tissues of *I. trifida*, ItfDA1-1 showed a low level of expression. The expression levels of six *ItfDA1*s (i.e., *ItfDA1-2*, *-3*, *-4*, *-5*, and *-7*) were high across all of the tissues. Furthermore, *ItfDA1-6* showed a low level of expression in all of the tissues and *ItfDA1-3* showed a low level of expression in the flowers and roots (Figure 6b).

### 2.6. QRT-PCR of the Sweet Potato DA1-like Gene in Different Tissues

**Figure 7:**
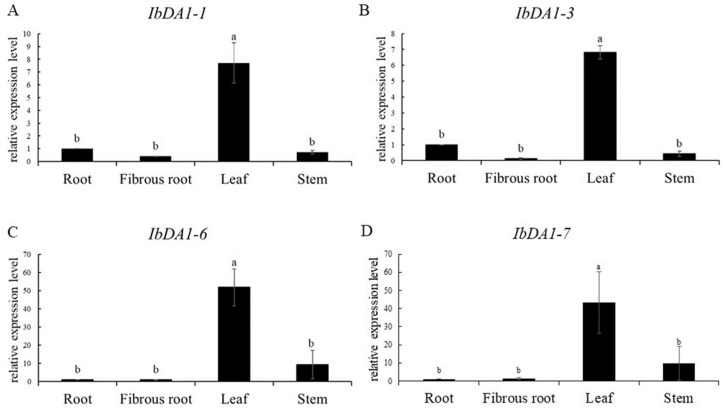
Analysis of *IbDA1* gene expression in different tissues of “HD7798” using qRT-PCR. Note: data are shown as mean ± SEM (*n* = 3). (**A**–**D**) Expression levels of *IbDA1-1* (**A**), *IbDA1-3* (**B**), *IbDA1-6* (**C**), and *IbDA1-7* (**D**) in roots, fibrous roots, leaves, and stems. Different letters means significant at 5% probability levels according to Duncan’s new multiple range method.

We analyzed the expression patterns of four genes (*IbDA1-1*, *IbDA1-3*, *IbDA1-6*, and *IbDA1-7*) in four different tissues to better understand the role of *IbDA1* in sweet potato growth and development (Figure 7). The gene expression was high in leaves, but there were no significant differences in the expression in the roots, fibrous roots, and stems. The expression of *IbDA1-1* was higher in the roots and fibrous roots than that of the other three genes.

## 3. Discussion

*AtDA1* is one of the smaller gene families in *Arabidopsis thaliana*, containing only eight genes. These genes not only play a regulatory role in grain size, but also play an important role in stress in *Arabidopsis thaliana* [33,34]. DA1 encodes a ubiquitination receptor, so DA1 may interact with the ubiquitination substrate in the ubiquitination protease pathway to promote its degradation. Recent studies have shown that the *DA1* gene family is involved in the ubiquitin protease pathway to regulate grain size [20,35]. At present, studies on the control of plant organ size by DA1 have been reported in a variety of crops, such as maize [17], rice [21], rape [15], wheat [22], and so on. These results indicate that DA1 has a relatively conservative function in crops and plays an important role in plant growth and development.

The *DA1* gene family members have been systematically analyzed in a variety of crops such as *Arabidopsis thaliana* [1], rice [20], rapeseed [15], maize [17], cotton [19], oat [18], and soybean [27], but the *DA1-like* genes of sweet potato have not been analyzed. With the rapid development of sweet potato sequencing technology and genetic engineering, it is convenient to mine candidate genes related to sweet potato yield and quality. Although the polyploid genome of sweet potato hindered the process of identifying grain size genes by mapping cloning, the use of the regulators of grain size identified in the model rice and *Arabidopsis thaliana* plants was effective as a candidate gene validation method. Therefore, this study systematically identified the *DA1-like* gene family in sweet potato, which provides a favorable theoretical and genetic reference for the subsequent study of *IbDA1* gene function.

In this study, we discovered *DA1-like* genes in sweet potatoes and their diploid relatives. Based on the evolutionary distance, the *DA1-like* genes were divided into three subgroups. According to the evolutionary tree, some DA1 proteins in sweet potatoes are closely related to OsDA1 proteins in rice and may have similar functions. These results suggest that DA1 proteins have differentiated significantly during long-term evolution; however, some DA1 proteins may still regulate organ size. According to the chromosomal location results, the sweet potato and its two diploid relatives have significantly different chromosomal distributions of *DA1-like* genes, suggesting the evolution of distinct variations among the species. In addition, we found that *IbDA1-7* and *IbDA1-8* are clustered in LG1; *IbDA1-4* and *IbDA1-5* are clustered in LG4; and *IbDA1-2* and *IbDA1-9* are clustered in LG11. Clustering has been extensively characterized in organisms such as fungi and plays an important role in transcriptional regulation mechanisms [36]. This suggests that clustering is conserved in Eukaryotes [37]. However, clustering exists only in sweet potatoes, but not in *I. batatas* and *I. trifida*, which is worth considering. We analyzed that the possible reason for this is that sweet potato has been artificially domesticated in the evolution of clustering so that its traits meet the needs of production. In the future, the functional verification of these cluster genes can be carried out to analyze the causes of the occurrence of these cluster genes. The physiological characteristics, phylogenetic relationships, gene structures, and promoter cis-elements of the *DA1* proteins were systematically studied. The expression patterns and qRT-PCR analyses suggested that *IbDA1-1* and *IbDA1-6* might regulate sweet potato traits.

### 3.1. Evolution of the DA1-like Gene Family in Sweet Potato and Its Two Diploid Relatives

The studied sweet potato and its two diploid relatives contained 27 *DA1*s, i.e., 9 *IbDA1*s, 11 *ItbDA1*s, and 7 *ItfDA1*s (Figure 1). These genes were relatively stable in terms of their structure and physical and chemical properties and had high functional conservation. Some genes had closer evolutionary relationships with rice *OsDA1*, such as *IbDA1-1*, *IbDA1-7*, and *IbDA1-6*. OsDA1 is homologous to AtDA1, has a high affinity for ubiquitin, and interacts with OsUBP15 to regulate rice grain size [20]. These results suggest that *IbDA1-1*, *IbDA1-7*, and *IbDA1-6* may regulate organ size in sweet potatoes. The *IbDA1* gene family in sweet potatoes comprises nine members, surpassing the numbers found in rapeseed, corn, oat, rice, and *Arabidopsis thaliana*; however, this is less than in cotton and soybean. In addition, more *DA1-like* genes were identified in *I. triloba* than in *I. trifida*, out of the two diploid wild relatives of sweet potatoes. The genome size may influence the diversity of *DA1-like* gene numbers and positions among species.

Over half of the DA1s contained ten conserved motifs. This indicates that these motifs have been relatively conserved (Figure 3a). The role of exons in gene diversity evolution is crucial [38]. Sweet potato and its two diploid relatives differ in the number of exons and introns in some DA1s. (Figure 3b). The Group I *IbDA1-1* genes contained 10 exons. This group’s homologous genes, *ItfDA1-1* and *ItbDA1-1*, contained 3 exons, and *IbDA1-2* possessed 13 exons, whereas *ItbDA1-5* contained the smallest number (only 2). These results suggest that DA1s evolved into genes with different functions in sweet potatoes and their diploid relatives. This is due to the differences in the structure of these exons.

### 3.2. Differences in the Functions of the DA1-like Gene Family in Growth and Development between Sweet Potato and Its Two Diploid Relatives

The regulation of plant organ size by *DA1-like* genes has been reported in several species [21,39,40,41] However, the function of *DA1-like* genes in regulating organ size in sweet potatoes has not been reported. We analyzed the cis-elements of these genes to explore the potential function of *DA1-like* genes in the growth and development of sweet potato and its two diploid relatives. As an extensively expressed transcription factor family in plants, the MYB transcription factor family actively participates in plant development and stress responses by binding specifically to MYB cis-elements in target gene promoters [42]. In this study, we found that the *DA1-like* promoter region contained MYB cis-acting elements in both the cultivated sweet potatoes and its two diploid relatives, indicating that the *DA1-like* gene family may interact with MYB transcription factors and thus participate in the growth, development, and stress resistance responses of sweet potatoes. In addition, we found that most of the *DA1-like* gene promoter regions contained many elements related to the response to hormonal and abiotic stresses. This provides a basis for studying the *DA1-like* gene family in sweet potatoes under stress conditions.

We focused on analyzing the expression pattern of DA1 in different tissues to further explore the potential function of *DA1-like* genes in the growth and development of sweet potato and its two diploid relatives. The results showed that the expressions of *IbDA1-2*, *IbDA1-4*, *IbDA1-5*, and *IbDA1-9* were downregulated in the eight tissues (stem, leaf, FR, ISR, RS, RB, DE, and PE). The stem showed high levels of *IbDA1-6* expression, and it was expressed in all the tissues. The expression levels of the *ItfDA1*s and *ItbDA1*s exhibited significant variation across six tissues in *I. trifida* and *I. triloba*. For instance, although *ItbDA1-1* was not detected in any tissue, *ItbDA1-2* was highly expressed in all of the tissues. These results suggest that *DA1-like* genes may play different roles in the growth and development of sweet potatoes. Furthermore, qRT-PCR was used to validate the expression profiles of DA1s across various sweet potato tissues. Interestingly, *IbDA1-1*, *IbDA1-3*, *IbDA1-6*, and *IbDA1-7* were highly expressed in the sweet potato leaves, indicating that they might be important functional genes involved in regulating sweet potato leaf development.

## 4. Material and Methods

### 4.1. Identification of DA1s

From the following websites: http://sweetpotato.plantbiology.msu.edu/ (accessed on 9 October 2023) and http://sweetpotato.plantbiology.msu.edu/ (accessed on 9 October 2023), we downloaded the genomic data of the sweet potato and two wild species. The protein sequences of the *DA1* gene family in *Arabidopsis thaliana* were obtained from the TAIR11 database (https://www.arabidopsis.org/, accessed on 9 October 2023), and the protein sequences of the *DA1* gene family in sweet potato and *Arabidopsis thaliana* (AT1G19270, AT4G36860, AT2G39830, AT5G66640, AT5G17890, AT5G66630, AT5G66620, and AT5G66610) were compared via BLAST. Meanwhile, Hidden Markov Model data of typical DA1 family protein knots were downloaded from the PFAM database (http://pfam.sanger.ac.uk, accessed on 10 October 2023), and the protein sequences containing characteristic domains were searched for using HMMER 3.0 software. The candidate proteins screened through the above two methods were merged, and multiple comparisons were performed using SnapGene 4.3.6 software. Incomplete or redundant sequences were removed manually, and screening results were checked using Pfam and SMART online analysis (http://smart.emblheidelberg.de/, accessed on 11 October 2023). Gene sequences without the *DA1* gene family domains or containing incomplete DA1 domains were removed, and the sweet potato *DA1* genes were finally obtained.

### 4.2. Distribution of DA1s on Chromosomes

Gene family members *IbDA1*, *ItfDA1*, and *ItbDA1* were located using annotation files for *I. batatas* genomes (https://ipomoea-genome.org/, accessed on 9 November 2023), *I. trifida*, and *I. triloba* (http://sweetpotato.plantbiology.msu.edu/, accessed on 9 November 2023).

### 4.3. Prediction of DA1 Protein Characteristics

We calculated DA1’s molecular weight, theoretical PI, instability index, and hydrophilicity with ExPASy (https://www.expasy.org/, accessed on 15 October 2023). Plant-mPLoc (http://www.csbio.sjtu.edu.cn/bioinf/plant-multi/, accessed on 15 October 2023) was used to predict DA1 subcellular localization.

### 4.4. Analysis of the Phylogenetic Relationships among DA1s

CLUSTALX 1.8.3 was used to perform multiple sequence alignments of the inferred amino acid sequences of the DA1s from *I. batatas*, *I. trifida*, *I. triloba*, *A. thaliana*, and *O. sativa*. After importing the alignment findings into MEGA11, 1000 bootstrap repeats and the greatest likelihood approach were used to build a phylogenetic tree (www.megasoftware.net, accessed on 19 November 2023). Then, using iTOL (http://itol.embl.de/, accessed on 19 November 2023), the phylogenetic tree was created.

### 4.5. Detection of Domains and Analysis of Conserved Motifs in DA1s

Using MEME 5.3.0 software (https://meme-suite.org/meme/, accessed on 21 November 2023), a conserved domain analysis of the *DA1*s was carried out, with a maximum motif count of 10. The conserved domain and exon–intron structures of the *DA1*s were visualized by applying Tbtools v2.056. PlantCARE was used to anticipate the *DA1* cis-elements.

### 4.6. Transcriptome Analysis

We used the published RNA-seq data of “Xushu 18” (PRJNA511028) to explore the expression pattern of *DA1*s in different tissues and organs of sweet potatoes [43]. From the Sweet Potato Genomics Resource (http://sweetpotato.plantbiology.msu.edu/, accessed on 25 November 2023), RNA-seq data for *ItfDA1*s and *ItbDA1*s were downloaded [26]. We plotted gene expression levels using TBtools.

### 4.7. Plant Material and qPCR

The sweet potato variety “HD7798”, independently bred by Hainan University, was utilized as the experimental material in this study, and four *DA1-like* genes were selected to analyze their expression patterns in leaves, stems, roots, and fibrous roots. *IbDA1* primers were produced using Primer 6.0 (Table S1). Reverse transcription was performed using an M-MLV RTase cDNA Synthesis Kit (TaKaRa, Kyoto, Japan). qRT–PCR was performed using the Roche LightCycler ^®^ 480II (Applied Biosystems, Foster City, CA, USA) System under the following conditions: 95 °C for 15 s, followed by 40 cycles of 95 °C for 15 s each, then 55 °C for 15 s, and 72 °C for 15 s. Relative quantification was performed using the 2^–∆∆Ct^ method [44].

### 4.8. Statistical Analysis

The student’s two-tailed t test was used for significant difference analysis between two samples. One-way ANOVA analyses followed by Tukey’s test (*p <* 0.05) were used for pairwise multiple comparisons. All of the analyses were performed using SPSS 24.0 software.

## 5. Conclusions

In this study, *DA1-like* genes are presented for the first time in sweet potato. Its two diploid relatives *I. trifida* and *I. triloba* were shown to have abundant genetic variation and diverse expression patterns, suggesting that the *DA1* gene family members may play different roles in the growth and development in these plants. This study provides a theoretical basis for further exploration of the function of the *DA1-like* genes in the growth and organ size regulation of sweet potatoes. However, further functional analyses are needed to clarify their role.

## Figures and Tables

**Table 1:** Characterization of *IbDA1* gene family members in sweet potato.

Gene ID	Gene Name	CDS (bp)	Protein (aa)	MW/kDa	pI	Phosphorylation Site			Aliphatic Index	GRAVY	Subcellular Locations
						Ser	Thr	Tyr			
g41815	*IbDA1-1*	1407	469	53.15	6.23	41	22	18	65.9	−0.607	nucleus
g46658	*IbDA1-2*	1992	664	74.59	7.49	52	30	26	85.41	−0.111	endoplasmic reticulum
g60449	*IbDA1-3*	1386	462	52.51	6.29	29	21	18	75.97	−0.413	nucleus
g15267	*IbDA1-4*	657	219	25.40	6.22	8	11	7	77.34	−0.2	cytoplasm
g15269	*IbDA1-5*	729	243	28.00	6.7	9	12	9	75.74	−0.147	cytoplasm
g17266	*IbDA1-6*	1614	538	61.55	5.09	52	23	21	64.49	−0.75	nucleus
g1178	*IbDA1-7*	1590	530	59.80	5.2	45	26	21	74.1	−0.45	nucleus
g1183	*IbDA1-8*	795	265	29.96	6.5	30	14	9	75.76	−0.422	nucleus
g46660	*IbDA1-9*	471	157	17.91	5.22	7	7	7	85.64	−0.049	cytoplasm

## Data Availability

All data and conclusions are included in this paper.

## Supplementary Materials

The following supporting information can be downloaded at: https://www.mdpi.com/article/10.3390/ijms25053000/s1.
