# Supplementary material for: Genome-Wide Identification and Expression Analysis of the DA1 Gene Family in Sweet Potato and Its Two Diploid Relatives
Source: Int J Mol Sci. 2024 Mar 5;25(5):3000. doi: 10.3390/ijms25053000 (PMC10931741; doi:10.3390/ijms25053000)
Supplement: Supplementary file 1 [file ijms-25-03000-s001.zip › ijms-2839288-supplementary.pdf]

Table S1. All primers used in this study.

Gene	F-Primer	R-Primer
IbDA1-1	TACTGGGCGTGGAAGTGGAT	TCGTTGCATAGGGGAAAGGTA
IbDA1-3	CGAGGAACAAACAGTCAGCAC	TTCACAGCGACGAGTCAATCTA
IbDA1-6	ATCATCGTCATCGTCCACCTC	TGAACCCATCGCCATAAGC
IbDA1-7	AGGGAAAGCGGTCTCAGTT	CCCATCACCATAAGCCACAG
